# Diagnosis and treatment pattern among rural and urban breast cancer patients in Southwest China from 2005 to 2009

**DOI:** 10.18632/oncotarget.11375

**Published:** 2016-08-18

**Authors:** Zuxiang Peng, Jia Wei, Xuesong Lu, Hong Zheng, Xiaorong Zhong, Weiguo Gao, Yunqin Chen, Jing Jing

**Affiliations:** ^1^ Department of Thyroid and Breast Surgery, Laboratory of Molecular Diagnosis of Cancer, State Key Laboratory of Biotherapy, National Collaborative Innovation Center for Biotherapy, West China Hospital, Sichuan University, Chengdu, China; ^2^ AstraZeneca R&D Information China, Zhangjiang Hi-Tech Park, Shanghai, China

**Keywords:** breast cancer, epidemiology, survival, treatment, real-world study

## Abstract

The incidence of all cancers in China is generally higher in urban areas; however, the mortality risk for affected patients is considerably higher in rural areas. We present a subanalysis investigating the differences in patient and disease characteristics, treatment patterns, and outcomes between rural and urban patients who were diagnosed with breast cancer at West China Hospital between 2005–2009. Baseline patient and disease characteristics were recorded, and patients were followed up for a minimum of 3 years, or until death. For this subanalysis, patients were stratified by their residential status (rural or urban). Of the 2252 patients in the cohort, 76.3% were from urban areas and 22.1% were from rural areas. Significant differences were observed in the prevalence of luminal A and human epidermal growth factor receptor 2-positive breast cancers among rural and urban patients. Estrogen receptor (ER)-positive patients were less likely to receive anti-ER therapy if they were from rural areas compared with urban areas; the use of aromatase inhibitors was also significantly lower for rural patients than urban patients. Univariate, multivariate, and Kaplan–Meier analyses all demonstrated that overall survival and progression-free survival were significantly lower for rural patients than urban patients.

## INTRODUCTION

China is the most populous country in the world [1]. In common with other emerging economies, China has experienced an increase in the reported incidence and prevalence of breast cancer [2]. In 2012, nearly 190,000 women were diagnosed with breast cancer [3]. In 2015, the incidence of breast cancer in China is expected to exceed 200,000 and to approach 230,000 by 2025 [3].

China has a heterogeneous population from a diverse range of socioeconomic and geographical backgrounds. One manifestation of this diversity is the difference in health status between populations from rural residential settings and those from urban residential settings [4]. Data suggest that the incidence and mortality rates of cancer (including breast cancer) vary between rural and urban populations [5]. During the 2005-2009 period, the incidence rate of breast cancer was reported to be approximately 2.3 times greater in urban areas than in rural areas [6]. Overall, the incidence of all cancers in China is generally higher in urban areas but the mortality risk is considerably higher in rural areas [7]. This may reflect higher exposure to risk factors in urban environments, and limited access to medical care and lower health education in rural areas.

The poorer prognosis for rural compared with urban patients is not solely explained by differences in the stage of disease at diagnosis. In a comparison of 1115 patients in China with early-stage breast cancer, overall survival (OS) and progression-free survival (PFS) were both significantly poorer for rural patients than urban patients [8]. Although this was a comparison of patients with early-stage breast cancer, rural patients were significantly more likely than urban patients to delay their initial visits to clinics for diagnosis [8].

Epidemiological and registry data can be important in the understanding of disease, treatment patterns and outcomes in populations of patients. In China, the national cancer registry is new and not yet fully established [2]. Thus, large regional patient registries are a valuable source of epidemiological information.

Previously, we have reported the treatment and survival patterns of 2252 patients diagnosed with breast cancer at West China Hospital, Sichuan University between 2005 and 2009 [9]. Here, we present subanalyses of the original dataset to investigate whether inequalities or disparities associated with patients' residential status (rural or urban) exist in the diagnosis, treatment, and long-term outcomes of patients with breast cancer.

## MATERIALS AND METHODS

### Ethical approval

This study was approved by the Clinical Test and Biomedical Ethics Committee of West China Hospital, Sichuan. Enrolled patients gave informed consent for their data to be collected, stored and analyzed.

### Patients

Female patients who received a diagnosis of breast cancer from the West China Hospital, Sichuan University, between the years of 2005 and 2009, inclusive, were recruited for this non-interventional study. Patients were followed up at least once every 4 months in the first 3 years after diagnosis. In the 3 to 5 years after diagnosis, the frequency of follow-up was reduced to once every 6 to 12 months. Annual follow-up was conducted for patients who had been diagnosed >5 years previously. Follow-up was conducted via interview at outpatient appointments or, if necessary, via telephone or postal contact. Lost to follow-up was defined as failure to make contact with the patient on >2 consecutive occasions.

The patient cohort, data collection, patient baseline characteristics (including age at diagnosis, menopause status, parity, breastfeeding history, treatment history and residential status), classification of tumor and molecular subtypes, and analyses of patient outcomes have previously been described elsewhere [9].

For these subanalyses, patients were stratified by their residential status. Cities in China are classified into four tiers, based on size and overall function (i.e., provincial capitals) [9]. Patients were categorized as urban if they lived in any city, regardless of tier, as rural if they lived in places other than cities, and as unknown if no information was recorded. Patients with unknown residential status were excluded from further analysis.

Baseline disease characteristics included molecular subtypes (luminal A, luminal B, human epidermal growth factor receptor 2 [HER^2^]-positive, or triple-negative breast cancer [TNBC]; Table S1), biomarkers (Estrogen receptor [ER], progesterone receptor [PgR], HER2, and Ki-67 status), and stage of disease.

Analysis of treatment patterns and associated outcomes included investigation of the use of endocrine therapies, which included anti-ER therapy (specifically, selective ER modulators [SERMs], selective ER down regulators [SERDs]), and aromatase inhibitors (AIs).

### Data analysis

The data described previously (including patient and disease characteristics, treatment patterns and outcomes) were subanalyzed in the context of the patients' residential status [9]. The primary endpoints were OS and PFS. PFS was defined as the interval between diagnosis and first disease progression or death, or was censored at the last follow-up. Disease progression was assessed by oncologists according to New Response Evaluation Criteria in Solid Tumors (RECIST version 1.1) [10]. Differences between rural and urban patients with respect to the primary endpoints were assessed using univariate and multivariate analyses. Differences were defined as statistically significant if *P* ≤ 0.05. Software used for statistical analyses included Bioconductor packages (Bioconductor; http://www.bioconductor.org) for R 3.1.3 (The R Foundation for Statistical Computing;http://www.r-project.org/) and GraphPad Prism 6 (GraphPad Software, Inc., CA, USA).

## RESULTS

### Patient baseline characteristics

In total, 2252 patients who were diagnosed with breast cancer at West China Hospital between 2005 and 2009 were included in the analysis. The majority of patients were classified as urban (76.3%), with the remainder classified as rural (22.1%) or unknown/uncategorized (1.6%). Patients with unknown or uncategorized residential status were excluded from further analysis. Baseline patient characteristics are summarized in Table 1. Median follow-up time for all patients was 4.60 years (range 0.01-8.43). Follow-up for urban and rural patients was comparable (urban 4.68 years [range 0.01-8.32]; rural 4.20 years [2.92-7.78]).

In summary, rural patients were significantly younger than urban patients at diagnosis and were also less likely than urban patients to be menopausal (both *P* < 0.001). Rural patients also had a history of multiple pregnancies, multiparity and breastfeeding (all *P* < 0.001) (Table 1).

**Table 1:** Baseline Characteristics Stratified by Residential Status

	Unknown *n* = 35 *n* (%)	Rural *n* = 498 *n* (%)	Urban *n* = 1719 *n* (%)	Total *n* = 2252 *n* (%)	*P*-value (difference between urban and rural)
**Age, years**					
Median (SD)	N/A	45.4 (±9.2)	50.4 (±11)	N/A	<0.001
≤48	19 (54.3)	336 (67.5)	775 (45.1)	1130 (50.2)	
>48	16 (45.7)	162 (32.5)	944 (54.9)	1122 (49.8)	
**Post-menopause**					
N/A	2 (5.7)	0 (0.0)	8 (0.5)	10 (0.4)	
No	23 (65.7)	363 (72.9)	932 (54.2)	1318 (58.5)	<0.001
Yes	10 (28.6)	135 (27.1)	779 (45.3)	924 (41.0)	
**Pregnancy Hx**					
N/A	10 (28.6)	128 (25.7)	273 (15.9)	411 (18.3)	
0	1 (2.9)	4 (0.8)	35 (2.0)	40 (1.8)	
1	8 (22.9)	40 (8.0)	292 (17.0)	340 (15.1)	<0.001
>1	16 (45.7)	326 (65.5)	1119 (65.1)	1461 (64.9)	
**Parity**					
N/A	10 (28.6)	128 (25.7)	272 (15.8)	410 (18.2)	
0	3 (8.6)	7 (1.4)	55 (3.2)	65 (2.9)	
1	17 (48.6)	187 (37.6)	979 (57.0)	1183 (52.5)	
>1	5 (14.3)	176 (35.3)	413 (24.0)	594 (26.4)	<0.001
**Breastfeeding Hx**					
N/A	0 (0.0)	9 (1.8)	15 (0.9)	24 (1.1)	
No	2 (5.7)	22 (4.4)	175 (10.2)	199 (8.8)	
Yes	33 (94.3)	467 (93.8)	1529 (88.9)	2029 (90.1)	<0.001
**Clinical stage**					
0	1 (2.9)	7 (1.4)	40 (2.3)	48 (2.1)	
I	7 (20.0)	65 (13.1)	316 (18.4)	388 (17.2)	
II	18 (51.4)	205 (41.2)	774 (45.0)	997 (44.3)	
III	6 (17.1)	153 (30.7)	373 (21.7)	532 (23.6)	
IV	0 (0.0)	5 (1.0)	41 (2.4)	46 (2.0)	
N/A	3 (8.6)	63 (12.7)	175 (10.2)	241 (10.7)	<0.001
**Early/late-stage**					
N/A	3 (8.6)	63 (12.7)	175 (10.2)	241 (10.7)	
Early (<IIIB)	28 (80.0)	347 (69.7)	1316 (76.6)	1691 (75.1)	
Late (≥IIIB)	4 (11.4)	88 (17.7)	228 (13.3)	320 (14.2)	0.008
**Subtype**					
N/A	6 (17.1)	88 (17.7)	272 (15.8)	366 (16.3)	
Luminal A	6 (17.1)	79 (15.9)	349 (20.3)	434 (19.3)	0.002
Luminal B	15 (42.9)	195 (39.2)	684 (39.8)	894 (39.7)	
HER2-positive	2 (5.7)	44 (8.8)	83 (4.8)	129 (5.7)	
TNBC	6 (17.1)	92 (18.5)	331 (19.3)	429 (19.0)	
**ER status**					
N/A	0 (0.0)	20 (4.0)	46 (2.7)	66 (2.9)	
Negative	11 (31.4)	181 (36.3)	568 (33.0)	760 (33.7)	
Positive	24 (68.6)	297 (59.6)	1105 (64.3)	1426 (63.3)	0.11
**PgR status**					
N/A	0 (0.0)	19 (3.8)	48 (2.8)	67 (3.0)	
Negative	12 (34.3)	200 (40.2)	661 (38.5)	873 (38.8)	
Positive	23 (65.7)	279 (56.0)	1010 (58.8)	1312 (58.3)	0.40
**HER2 status**					
N/A	1 (2.9)	25 (5.0)	63 (3.7)	89 (4.0)	
Negative	30 (85.7)	404 (81.1)	1493 (86.9)	1927 (85.6)	
Positive	4 (11.4)	69 (13.9)	163 (9.5)	236 (10.5)	0.004
**Ki-67 status**					
N/A	4 (11.4)	69 (13.9)	189 (11.0)	262 (11.6)	
<14%	12 (34.3)	130 (26.1)	533 (31.0)	675 (30.0)	
≥14%	19 (54.3)	299 (60.0)	997 (58.0)	1315 (58.4)	0.08

ER = estrogen receptor, HER2 = human epidermal growth factor receptor 2, Hx = history, N/A = not applicable or available. PgR = progesterone receptor, SD = standard deviation, TNBC = triple-negative breast cancer.

### Disease baseline characteristics

The proportion of patients with luminal A tumors was significantly lower for rural patients (15.9%) compared with urban patients (20.3%; *P* = 0.002, Table 1). HER2-positive breast cancer was higher in patients from rural (8.8%) than urban areas (4.8%), although this difference was not significant. The incidence of both luminal B (rural: 39.2%, urban: 39.8%) and TNBC (rural: 18.5%, urban: 19.3%) were comparable in patients from rural and urban areas. There were no significant differences in the proportion of rural and urban patients according to ER-positivity, PgR-positivity, or Ki-67 >14% (Table 1).

For disease stage, defined as early-stage disease if the clinical stage was judged to be at most IIIa and late-stage disease if the clinical stage was judged to be IIIb or IV, patients from rural areas were significantly more likely than patients from urban areas to present with late-stage disease (rural: 17.7%, urban: 13.3%; *P* = 0.008, Table 1).

**Table 2:** Differences Between Endocrine Therapy Treatments Received by Urban and Rural Patients According to Estrogen Receptor and Progesterone Receptor Biomarker Status

	All patients					
	ER-positive			PgR-positive		
	Rural	Urban	*P*	Rural	Urban	*P*
Patients, *n*	297	1105	<0.001	279	1010	<0.001
Received any endocrine therapy, *n* (%)	246 (82.8)	1025 (92.8)	<0.001	224 (80.3)	916 (90.7)	<0.001
Received SERMs, *n* (%)	180 (60.6)	602 (54.5)	0.007	168 (60.2)	567 (56.1)	0.05
Received AI treatment, *n* (%)	84 (28.3)	604 (54.7)	<0.001	69 (24.7)	522 (51.7)	<0.001

AI = aromatase inhibitors, ER = estrogen receptor, PgR = progesterone receptor, SERM = selective estrogen receptor modulator.

### Treatment Patterns

#### Endocrine therapy

Regardless of disease stage, ER-positive rural patients were significantly less likely than urban patients to receive treatment with endocrine therapy (rural: 82.8%, urban: 92.8%; *P* < 0.001) (Table 2). A similar observation was made for patients with PgR-positive tumors (rural: 80.3%, urban: 90.7%; *P* < 0.001) (Table 2). For patients who were ER-positive and received anti-ER treatment (rural: *n* = 180, urban: *n* = 602), mean anti-ER treatment length was significantly longer for rural patients than for urban patients (1417.7 vs 1275.2 days; *P* = 0.02).

In patients with ER-positive tumors, treatment with AIs was significantly lower in rural patients compared with urban patients (rural: 28.3%, urban: 54.7%; *P* < 0.001). Rural patients were also less likely than urban patients to receive AIs for a period of 5 years or more (rural: 4.2%, urban: 13.8%; *P* < 0.001).

A minority of both rural and urban patients who received anti-ER therapy were ER-negative, and the level was similar between patients from both residential settings (rural: 14.3%, urban: 12.4%; *P* = 0.48).

#### Chemotherapy

While the majority of patients (>90%) from both rural and urban areas received chemotherapy, the exposure was significantly higher in rural patients than urban patients (rural: 93.4%, urban: 90.9%; *P* < 0.001).

Rural patients were more likely to receive treatment with anthracyclines (specifically epirubicin) than urban patients (rural: 71.7%, urban: 65.2%; *P* = 0.007) and were also more likely than urban patients to receive 5-fluorouracil + epirubicin + cyclophosphamide ± paclitaxel (FEC±T) (rural: 31.7%; urban: 22.7%). Both anthracycline plus taxane and FEC plus taxane treatment were significantly increased in rural patients compared with urban patients, regardless of early- or late-stage disease (Table 3). In contrast, treatment with 5-fluorouracil was higher in urban patients (rural: 36.3%, urban: 28.9%; *P* = 0.002). Taxane use was comparable for both early- and late-stage disease, regardless of residential status (Table 3).

#### Radiotherapy

Radiotherapy was significantly increased in both early- (33.6% vs 27.1%) and late-stage disease (70.2% vs 55.7%) for urban patients compared with rural patients (Table 3).

**Table 3:** Differences in Chemotherapy and Radiotherapy received by Urban and Rural Patients According to Early- and Late-Stage Disease

	Early-stage			Late-stage			All patients		
	Rural	Urban	*P*	Rural	Urban	*P*	Rural	Urban	*P*
Patients, *n*	347	1316		88	228		498	1719	
Received radiotherapy, *n* (%)	94 (27.1%)	442 (33.6%)	0.024	49 (55.7%)	160 (70.2%)	0.017	165 (33.1%)	660 (38.4%)	0.035
Received taxane alone, *n* (%)	32 (9.2%)	141 (10.7%)	0.489	5 (5.7%)	29 (12.7%)	0.103	42 (8.4%)	188 (10.9%)	0.113
Received anthracycline without taxane, *n* (%)	111 (32.0%)	325 (24.7%)	0.007	19 (21.6%)	21 (9.2%)	0.005	155 (31.1%)	390 (22.7%)	0.0002
Received FEC ± T treatment, *n* (%)	103 (29.7%)	315 (23.9%)	0.031	28 (31.8%)	29 (12.7%)	0.0002	158 (31.7%)	390 (22.7%)	0.00006

C = cyclophosphamide, E = epirubicin, F = 5-Fluorouracil, T = paclitaxel.

### Survival

**Figure 1:**
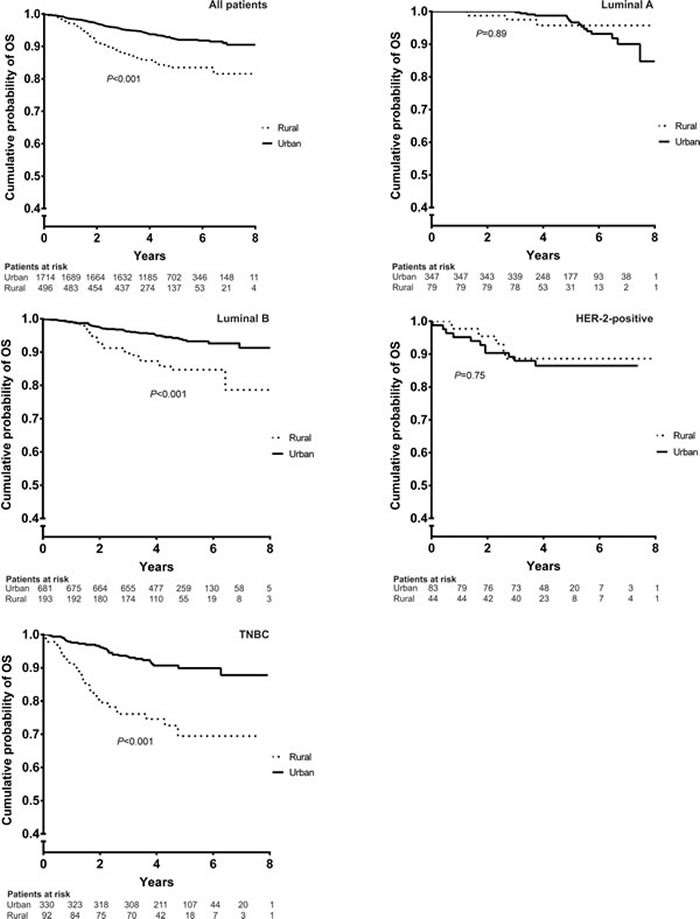
Kaplan-Meier analysis of OS (A) and PFS (B) for rural and urban patients HER2 = human epidermal growth factor positive, OS = overall survival, PFS = progression-free survival, TNBC = triple-negative breast cancer.

Univariate analysis demonstrated that urban patients had significantly greater OS and PFS than rural patients; these observations were subsequently confirmed using multivariate analysis (OS: HR 0.42 [95% CI 0.32-0.56], *P* < 0.001; PFS: HR 0.63 [95% CI 0.47-0.85], *P* = 0.002) and further supported by Kaplan-Meier analyses of OS (Figure 1A) and PFS (Figure 1B). The residential effect was not significant for patients with luminal A and HER2-positive breast cancer.

**Figure 2:**
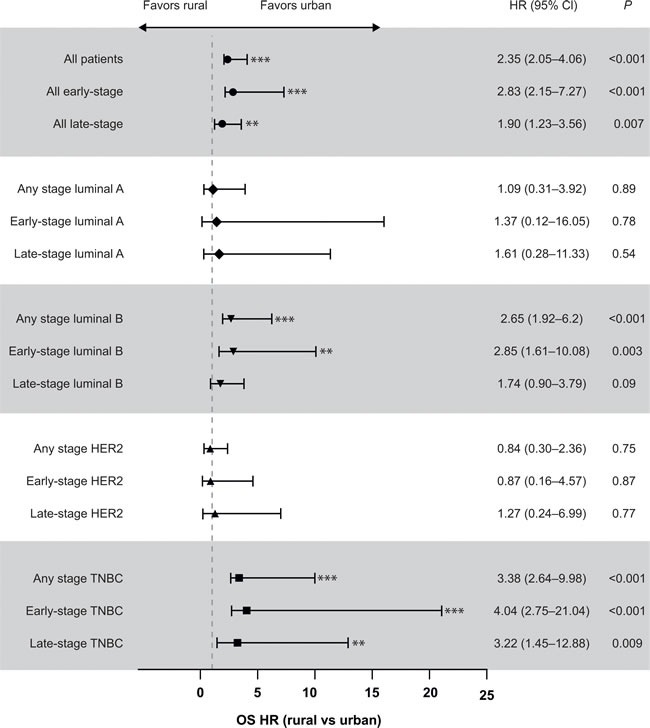
Analysis of the effect of patients' residential status (rural or urban) on OS (A) and PFS (B) by disease stage and breast cancer subtype *P < 0.05; **P < 0.01; ***P < 0.001. CI = confidence interval, HER2 = human epidermal growth factor receptor 2, HR = hazard ratio, OS = overall survival, PFS = progression-free survival, TNBC = triple-negative breast cancer.

Residential status had a significant effect on PFS in patients with early-stage disease (Table 4). Subanalyses demonstrated that residential status had a significant effect on OS and PFS for all luminal B and TNBC, regardless of disease stage (result not significant for late-stage luminal B) (Figures 2A and 2B). Univariate analysis also demonstrated significantly increased OS (HR 0.40 [95% CI 0.20-0.80], *P* < 0.001) and survival after metastasis (HR 0.34 [95% CI 0.16-0.72], *P* = 0.005) for pre-menopausal patients from urban areas compared with rural areas (Table 5). Significantly increased metastasis-free survival was also reported in post-menopausal patients from urban areas compared with rural patients (HR 0.54 [95% CI 0.30-0.97], *P* = 0.04) (Table 5).

**Table 4:** Univariate Analysis of the Effect of Patients' Residential Status (Rural or Urban) on Outcomes in Early- and Late-Stage Breast Cancer (All Subtypes)

	Early-stage		Late-stage	
	Rural (*n*= 347)	Urban (*n*= 1316)	Rural (*n*= 88)	Urban (*n*= 228)
**OS**				
Events, *n* (%)	35 (10.1)	55 (4.2)	36 (40.9)	59 (25.9)
HR (95% CI)	0.37 (0.24–0.56)		0.53 (0.34–0.80)	
*P*	< 0.001		0.003	
**PFS**				
Events, *n* (%)	47 (13.5)	113 (8.6)	45 (51.1)	95 (41.7)
HR (95% CI)	0.57 (0.40–0.81)		0.77 (0.54–1.10)	
*P*	0.002		0.16	

CI = confidence interval, HR = hazard ratio, OS = overall survival, PFS = progression-free survival.

**Table 5:** Survival Benefit for Urban Patients, Compared with Rural Patients, According to Menopausal Status

	HR	95% CI	*P*
**Pre-menopausal**			
OS	0.4047	0.20–0.81	< 0.001
MFS	0.79	0.49–1.3	0.34
RFS	0.43	0.17–1.1	0.06
Survival after metastasis	0.34	0.16–0.72	0.005
PFS	0.74	0.48–1.1	0.17
**Post-menopausal**			
OS	0.62	0.29–1.3	0.22
MFS	0.54	0.30–0.97	0.04
RFS	1.3	0.3–5.6	0.73
Survival after metastasis	1	0.42–2.6	0.93
PFS	0.67	0.38–1.2	0.18

CI = confidence interval, HR = hazard ratio, MFS = metastasis-free survival, OS = overall survival, PFS = progression-free survival, RFS = relapse-free survival.

### Treatment and survival

Urban patients with ER-positive tumors who received endocrine therapy had significantly better OS than rural patients (HR 0.53 [95% CI 0.30-0.96], *P* = 0.04), although there was no difference in PFS. Similarly, for patients with ER-positive tumors who received AIs, urban patients had significantly better OS than rural patients (HR 0.17 [95% CI 0.04-0.73], *P* = 0.02), with no difference in PFS.

For patients receiving paclitaxel, either alone or in combination, there were clear outcome differences between rural and urban patients. Improved PFS for luminal B and TNBC subgroups of urban patients, compared with rural patients, was observed with paclitaxel-containing regimens (Table 6).

**Table 6:** Progression Free Survival Benefit for Urban Patients, Compared with Rural Patients, Among Patients Receiving Taxane Alone or in Combination

Regimen/subgroup	HR (95% CI)	*P*
**Taxane alone**		
Luminal B (any-stage)	0.49(0.32-0.77)	0.002
Luminal B (early-stage)	0.40(0.19-0.83)	0.01
TNBC (any-stage)	0.44 (0.26-0.76)	0.003
TNBC (early-stage)	0.37 (0.17-0.79)	0.01
TNBC (late-stage)	0.50 (0.19-1.30)	0.16
**Taxane + anthracyclines**		
Luminal B (any-stage)	0.51 (0.31-0.83)	0.006
TNBC (any-stage)	0.42 (0.23-0.79)	0.007
TNBC (late-stage)	0.42 (0.15-1.17)	0.1
**Taxane + cyclophosphamide**		
TNBC (any-stage)	0.40 (0.19-0.85)	0.02
TNBC (late-stage)	0.18 (0.35-0.93)	0.04

CI = confidence interval, HR = hazard ratio, TNBC = triple-negative breast cancer.

Taxane includes paclitaxel (domestic), paclitaxel (import), paclitaxel albumin, paclitaxel liposome, docetaxel (domestic), docetaxel (import); Anthracyclines include doxorubicin, epirubicin (domestic), epirubicin (imported), pirarubicin and mitoxantrone.

## DISCUSSION

The majority of patients in our cohort originated from urban areas, with only 22.1% of patients identifying as rural inhabitants. OS and PFS were significantly better in patients from urban areas than in patients from rural areas. This relative OS and PFS benefit for urban patients over rural patients was observed in univariate, multivariate, and Kaplan-Meier analyses. These results are comparable to studies from other countries which have shown patients with breast cancer from rural areas have poorer outcomes compared with patients from urban areas [11-15].

Patients from rural areas were significantly more likely than urban patients to present with late-stage disease. This finding may be have a number of contributing factors [8]. For example, disparities in health services between rural and urban areas may result in longer waits for initial medical investigation and referral for diagnosis for rural patients.

Socioeconomic factors may also contribute to the differences in patient outcomes [16, 17]. Poor rural patients may be less able to afford out-of-pocket treatment expenses, leading to a delay in visiting a doctor until the disease is advanced. Additionally, rural patients may not have health insurance that would pay for mammograms, making early diagnosis more difficult [6]. Furthermore, rural patients may feel less able than urban patients to take the time away from daily activities and work to attend medical appointments. Geographical and travel issues may also contribute to delayed diagnosis in rural patients, as travel to medical centers can be a greater logistical challenge.

Differences in health education between rural and urban patients may also play a role determining outcome. Awareness and knowledge of breast cancer is much higher in urban than rural areas, and patients in urban areas are more likely to be aware of breast cancer signs and symptoms [18]. The differences in OS and PFS between rural and urban patients suggest that improving awareness and education of breast cancer in rural areas may be a strategy for improving outcomes in patients.

In this cohort, the luminal A subtype was found to be more prevalent in the urban breast cancer population than the rural breast cancer population (*P* = 0.002). This may partially account for the better prognosis for urban patients compared with rural patients, as luminal A subtype is associated with better survival than other breast cancer subtypes [19, 20].

Patients with ER-positive tumors from rural areas were significantly less likely to receive endocrine therapy than patients from urban areas and were also less likely to receive AIs than urban patients. Where rural patients did receive AIs the mean treatment duration was significantly shorter than that for urban patients and AI treatment was strongly associated with a better prognosis. A possible explanation for this observation is that rural patients may present with later-stage and more advanced breast cancer that may have required treatment with chemotherapy rather than anti-ER therapy. Furthermore, increased rates of late-stage disease may have led to increased rates of palliative care in rural patients compared with urban patients.

Patients in our cohort were more likely to receive antimetabolites if they lived in rural rather than urban areas. Antimetabolites are a relatively inexpensive option as they are off-patent. In addition to cost considerations, some of the observed differences in the proportion of rural and urban patients receiving particular therapeutics may also be attributable to the higher proportion of rural patients than urban patients with advanced/late-stage disease and differences in the proportion of breast cancer subtypes between the two patient populations.

Our study does have some limitations. Having been collected from a single hospital in southwest China, our data may not apply to other regions. Follow-up interviews may have been confounded by patients' recall errors or by patients having misunderstood their medical treatment. Patient adherence to treatment was also not assessed in this study so it cannot be discounted that lack of compliance rather than residential status may have affected some of the results reported here. Finally, as HER2 testing methods and technologies are relatively new to the region, the reliability of HER2 testing in our cohort is unknown.

In conclusion, we have presented data that add to the existing evidence of a difference in breast cancer outcomes in rural and urban Chinese populations. With rural patients being more likely to be diagnosed with late-stage, advanced disease, we believe that this highlights the importance of improving awareness and education of breast cancer to allow patients to identify signs and symptoms early. Measures should be taken to reduce the disparity in diagnostic facilities that exists between rural and urban patients. Finally, treatment practices and decisions should be standardized and efforts made to improve physicians' knowledge of existing guidelines, to ensure that all patients receive the optimal treatment regardless of where they live.

## Abbreviations

AI: aromatase inhibitor
CI: confidence interval
ER: estrogen receptor
FEC: fluorouracil + epirubicin + cyclophosphamide
HER2: human epidermal growth factor receptor 2
HR: hazard ratio
OS: overall survival
PFS: progression-free survival
PgR: progesterone receptor
SD: standard deviation
SERD: selective estrogen receptor degrader
SERM: selective estrogen receptor modulator
T: paclitaxel
TNBC: triple-negative breast cancer
